# Dengue Fever Associated Haemophagocytic Lymphohistiocytosis: A Report of Two Children

**DOI:** 10.7759/cureus.11232

**Published:** 2020-10-29

**Authors:** Vijayakumary Thadchanamoorthy, Kavinda Dayasiri

**Affiliations:** 1 Clinical Sciences Department, Eastern University, Faculty of Health Care Sciences, Batticaloa, LKA; 2 Pediatrics, Base Hospital Mahaoya, Mahaoya, LKA

**Keywords:** dengue haemorrhagic fever, splenomegaly, hyperferritinemia, hypertriglyceridemia, hemophagocytic lymphohistiocytosis (hlh)

## Abstract

Hemophagocytic lymphohistiocytosis (HLH) is a rare, fatal complication of dengue infection and often leads to multi-system involvement and failure. Early recognition is crucial in improving the outcome. We report two children who developed secondary haemophagocytic lymphohistiocytosis following dengue fever. A 14-year-old girl who was diagnosed with beta-thalassemia major presented with dengue hemorrhagic fever and developed a continuous very high fever, persistent thrombocytopenia, and anemia despite several transfusions of blood and blood products. The other child was a 12-year old girl who, following initial confirmation of dengue fever, presented with persistent fever and hepatosplenomegaly. The diagnosis of HLH in both children was confirmed by clinical and laboratory features supported by the demonstration of haemophagocytes in the bone marrow. Both children were treated with steroids and supportive care and made a gradual recovery with treatment. Second-line immune-suppressive treatment was not needed. Whilst sepsis is a priority differential diagnosis in children with persistent fever following recovery from dengue fever, HLH should always be suspected early in these patients. Early, appropriate immunosuppressive treatment is likely to improve long-term outcomes and prevent mortality.

## Introduction

Dengue infection is a mosquito-borne, endemic, neglected tropical disease caused by one of four dengue virus subtypes, mainly in tropical and subtropical countries. Dengue fever is considered a serious health problem worldwide [1]. Secondary hemophagocytic lymphohistiocytosis (HLH) is a potentially fatal condition that develops following several primary causes, including dengue. Delayed diagnosis and treatment of HLH are often associated with high mortality and morbidity. Thus early recognition is important for early, appropriate curative therapy. HLH is now being increasingly detected in clinical practice due to improved knowledge and awareness amongst clinicians [2]. There is still much work to be done to raise awareness, improve treatment options, and long-term outcomes of this complex condition [3, 4]. We report two children who developed dengue infection-associated HLH. Both children had a successful recovery following early treatment with steroids and supportive care. 

## Case presentation

Case 1

A 14-year-old patient diagnosed with thalassemia major presented with continuous fever (highest 39.8° C) and abdominal pain for five days. She was initially treated for urinary tract infection by the general practitioner since she had high white blood cells with a neutrophil predominance (white blood cells [WBC]: 12x103, neutrophils: 80%, hemoglobin: 9g/dl; platelet: 275x103), high C-reactive protein (CRP: 12 mg/dl) and urine full report showed a field full of pus cells pending urine culture. She had a history of recurrent urinary tract infections. Her oral intake and sleep had been poor during the two days before admission. She had not passed urine for 12 hours. Following admission, she passed blood-stained stools. Her previous record revealed that she had 4 cm hepatomegaly and 4 cm splenomegaly on discharge following routine blood transfusions the previous week, with the post-transfusion hemoglobin being 11.9 mg/dL.

On examination, she had average body weight (34 kg), looked ill, irritable with congested conjunctiva, cold extremities, bleeding from puncture sites, and showed mild generalized body swelling. The abdomen was tender and physical examination revealed hepatomegaly of 8 cm and splenomegaly of 11 cm. Blood pressure was 80/60 mmHg, and the pulse rate was 150 beats per minute with low volume. Other systems examination had been clinically normal. While awaiting investigations, the point-of-care ultrasound revealed moderate amounts of pleural effusions and ascites. A provisional diagnosis of dengue hemorrhagic fever with secondary bacterial infection (urinary tract infection) was made, and she was resuscitated with intravenous fluids and other supportive management. She was also started intravenous cefotaxime and needed admission to the intensive care unit (ICU) for ventilation and further management.

Investigations revealed white blood count - 14x103/cumm, neutrophils - 68%, platelets - 85x103/cumm, haemoglobin - 8 g/dl, alanine transaminase (ALT) - 245 U/L, aspartate transaminase (AST) - 285 U/L, random blood sugar - 6 mmol/l and C-reactive protein - 24 mg/dL. The renal function had been normal, and urine output was 0.4 ml/kg/hour. Blood culture showed no growth, and urine culture revealed pseudomonas sensitive to ceftazidime. The chest X-ray was normal except for pleural effusion. An ultrasound scan of the abdomen done by a consultant radiologist showed gross hepatomegaly with normal kidneys and associated ascites. She was managed with blood and platelet transfusion and needed inotropes for maintaining hemodynamics while receiving ventilator care in addition to changing the antibiotic to ceftazidime. Both immunoglobulin G (IgG) and immunoglobulin M (IgM) dengue antibodies were positive on the seventh day of febrile illness.

**Figure 1:**
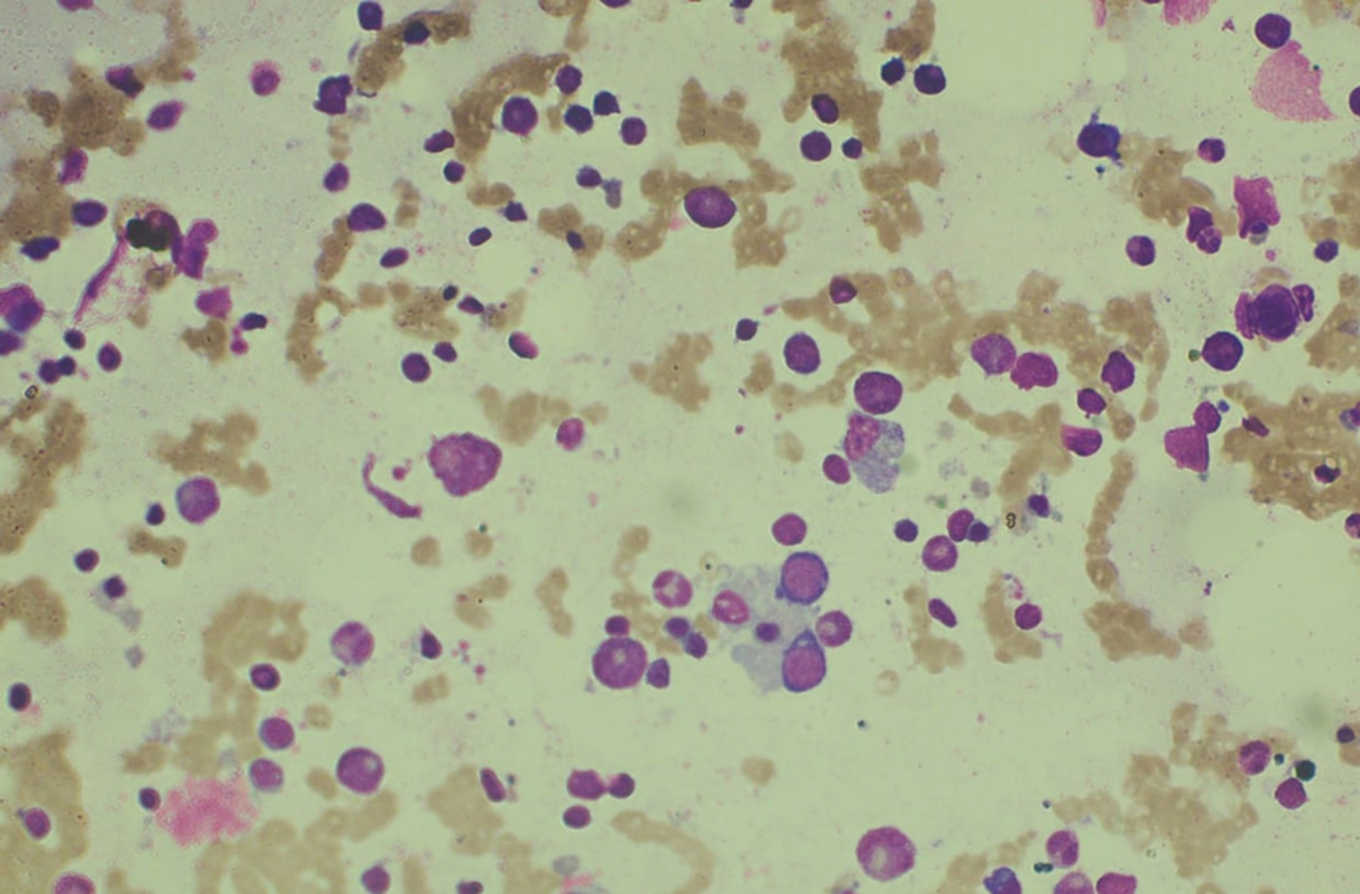
Bone marrow showing multiple hemophagocytes

Although she recovered from dengue and septic screening was normal, she continued to have a very high fever, persistent thrombocytopenia, and anemia despite several transfusions of leucocyte reduced washed blood and blood products. Investigations were repeated, as shown in Table 1. The investigations showed high ferritin, increased triglycerides, and bicytopenia. At this stage, she was suspected of having haemophagocytic lymphohistiocytosis, and the diagnosis was confirmed by bone marrow examination (Figure 1) and supportive clinical and laboratory features. She was commenced on intravenous dexamethasone and immunoglobulin. She recovered on day 14 of illness with normalization of investigations findings.

**Table 1:** Investigation results of case 1 and case 2 SGOT - serum glutamic oxaloacetic transaminase; SGPT - serum glutamic pyruvic transaminase; ESR - erythrocyte sedimentation rate; CRP - C-reactive protein; LDH - lactate dehydrogenase; APTT - activated partial thromboplastin time

Investigation	Value for case 1	Value for case 2	Reference range
Serum albumin	22 g/l	26 g/l	34 - 50 g/l
SGOT	1024 U/L	692 U/L	10 - 40 U/L
SGPT	642 U/L	387 U/L	10 - 40 U/L
ESR	20/hour	12 1^st^ hour	<20 1^st^ hour
CRP	<6	4 mg/l	<5 mg/l
Serum bilirubin - total	64 µmol/l	44 µmol/l	3 - 20
Serum bilirubin - direct	24 µmol/l	21 µmol/l	<3
Blood picture	Target cell, nucleated red blood cell, no abnormal cells	No morphological abnormalities	
Serum ferritin	32,000	6,000	24 - 336 microgra/l
Serum fibrinogen	124 mg/dL	118 mg/dL	200 - 400 mg/dL
Serum sodium	131 meq/l	129 meq/l	135 - 145 meq/l
Serum triglycerides	284 mg /dL	251 mg/dL	Less than 150 mg/dL
Serum LDH	1280 U/L	2106 U/L	140 - 280 U/L
Prothrombin time	13 seconds	12 seconds	10 - 14 seconds
APTT	32 seconds	34 seconds	25 - 35 seconds
Hemoglobin	8 g/dl	9.9 g/dL	11.5 - 13 g/dL
Platelets	44x10^3^	45x10^3^	150 - 450 x10^3^
Leucocytes	4,000	6,300	4,000 - 10,000
Neutrophils	25%	45%	45 - 75%

Case 2

A 12-year-old girl diagnosed with trisomy 21 was referred for evaluation of continuing fever for ten days. The child initially had a moist cough associated with fever. On the seventh day, she presented to the local hospital with continuing fever associated with gum bleeding, melena, and generalized macular, blanching erythematous rash. She was subsequently found to have thrombocytopenia (13x103/cumm) associated with leucopenia (WBC: 3x103/cumm, neutrophils - 60%). Liver functions were deranged (serum glutamic oxaloacetic transaminase [SGOT**] **- 966 U/L, serum glutamic pyruvic transaminase​​​​​​​ [SGPT**] **- 489 U/L). Dengue IgM was positive, and IgG was negative. Focused USS did not reveal evidence of fluid extravasation, and she was managed as for dengue fever and was hemodynamically stable. 

But the child continued to have a fever despite having a dengue recovery rash and negative septic screen, and she was referred for further evaluation. Physical examination at the tertiary hospital revealed hepatomegaly (8 cm), splenomegaly (7 cm), and macular rash evolving to a petechial rash. Investigations revealed hypoalbuminemia, direct hyperbilirubinemia, hyperferritinemia, and hypertriglyceridemia (see Table 1). She was clinically suspected as haemophagocytic lymphohistiocytosis and confirmed with bone marrow examination, which revealed normal cellularity with reactive changes and megaloblastic erythropoiesis. The increased histiocytic activity was seen with haemophagocytosis. There was no evidence of hematological or non-hematological malignancy. She was treated with steroids and supportive care and made a gradual recovery with treatment. Second-line immune-suppressive treatment was not needed. 

## Discussion

HLH is an uncommon inflammatory disease that carries major therapeutic difficulties, and the diagnosis is often challenging to the clinicians. It is characterized by the activation of macrophages that cause phagocytosis of blood cells in the bone marrow. HLH may be primary (familial) or secondary. Secondary HLH is due to over activation of the immune system. There are several causes for secondary HLH, including a variety of viral, bacterial, fungal, and parasitic infections, as well as collagen vascular diseases and malignancies, particularly T-cell lymphoma [5]. The diagnosis of HLH is based on the presence of five out of eight criteria (fever, splenomegaly, hyperferritinemia, bicytopenia, hypertriglyceridemia and/or hypofibrinogenemia, and hemophagocytosis, absent natural killer (NK) cell activity, and high-soluble interleukin-2-receptor levels) [6-8]. Both children presented in our case had fulfilled the criteria for HLH syndrome.

Phagocytosis of blood cells and their precursors is a symbol of hemophagocytic syndromes, which are mostly caused by the activation of monocytes and macrophages. Over-activation of macrophages and monocytes in HLH is due to stimulation by high levels of activating cytokines, including interferon-γ, soluble interleukin-2 receptor, tumor necrosis factor (TNF-α), interleukin-1, and Interleukin-6 [5]. Dengue is a rare case of HLH. Virus-infected T cells secrete cytokines like TNF-α and interferon-gamma (IFN-γ), which probably account for the development of HLH syndrome [9]. There are previous reports which describe HLH associated with dengue hemorrhagic fever (DHF) [10]. Secondary HLH and severe sepsis, systemic inflammatory response syndrome (SIRS), multiple organ dysfunction syndromes (MODS) have common clinical and laboratory inflammatory phenotypes. Progressive pancytopenia, along with the presence of hemophagocytes in bone marrow, guide confirmation of the diagnosis of HLH. Case 1 was brought to the hospital late with unusual presentation having features of invasive bacterial infection with urinary and gastrointestinal symptoms. This was the reason that the GP missed the diagnosis. As she had thrombocytopenia, living in endemic to dengue, pleural effusion in the ultrasound, and dengue serology, the child was diagnosed accurately as having DHF, and later bone marrow confirmed HLH following the development of unusual features which were suggestive of HLH.

Treatment of HLH includes treatment of the underlying cause, supportive, and immune suppressive therapy. Specific treatment of HLH includes dexamethasone or methylprednisolone, etoposide, and intrathecal methotrexate. Intravenous immunoglobulin has been used in a few cases, either alone or in combination with dexamethasone or methylprednisolone. In addition, antithymocyte globulin or alemtuzumab, anti-interferon-γ monoclonal antibodies, and hematopoietic stem cell transplantation are available other modalities for HLH treatment [11]. The complexity of the clinical condition, delayed admission, shock on admission, difficulty in differentiation from sepsis, and multi-organ dysfunction often leads to delayed diagnosis of HLH in children with dengue fever. Therefore, it is crucial to have a high index of clinical suspicion and investigate children with unusual yet supportive clinical features of HLH without delay to improve outcomes. 

## Conclusions

Whilst sepsis is a priority differential diagnosis in children with persistent fever following recovery from dengue fever, HLH should always be suspected early in these patients. Early recognition and prompt institution of appropriate immunosuppressive therapy is the most vital factor for achieving good prognosis.
